# Comparison of Gas Adsorption Properties in Methylated and Non-Methylated Imine-Linked Nanoporous Covalent Organic Frameworks

**DOI:** 10.1021/acsanm.5c02616

**Published:** 2025-09-15

**Authors:** Stijn Paulusma, Thijmen A. van Voorthuizen, Hans-Gerd Janssen, Louis C. P. M. de Smet

**Affiliations:** † Laboratory of Organic Chemistry, Wageningen University Stippeneng 4, 6708 WE Wageningen, The Netherlands; ‡ Unilever Foods Innovation Centre — Hive, Bronland 14, 6708 WH Wageningen, The Netherlands

**Keywords:** porous nanomaterials, methyl groups, gas adsorption, specific probes, inverse gas chromatography

## Abstract

Gas-material interactions are crucial in various industrial processes, including microchip fabrication, fuel production, and exhaust gas treatment. Covalent organic frameworks (COFs) are a class of porous, crystalline nanomaterials composed of organic building blocks linked by strong covalent bonds. Their highly tunable surface properties make them promising candidates for gas adsorption. In this study, we explored how the presence of methyl groups influences the gas adsorption properties of volatile organic compounds, i.e., probes, in stable, imine-linked COFs. Enthalpy measurements revealed that Me_3_TFB-BD, a methylated COF, exhibited weaker interactions with toluene (−41.3 kJ/mol) and heptane (−45.6 kJ/mol) compared to its nonmethylated derivative TFB-BD (−50.5 kJ/mol and −54.0 kJ/mol, respectively). Partition coefficient (*K*) data also indicated that TFB-BD has stronger interactions with a broader set of specific probes than Me_3_TFB-BD, likely due to a higher imine bond accessibility. Both COFs also showed strong interactions with polar alcohol probes, which can be attributed to their high polarizability. Analysis of Me_3_TFB-PA, a COF with a lower methyl to carbon ratio, led to further reduction in the COF-probe interaction strength. All three COFs demonstrated moderate adsorption capacities, though TFB-BD showed the highest uptake for toluene (0.1 μmol/m^2^) and heptane (∼0.07 μmol/m^2^), due to its stronger interactions and smaller pore size. Additionally, selectivity analysis revealed that TFB-BD exhibited the strongest affinity for a broad range of probes. Overall, this study highlights the potential of COFs as tunable and promising materials for targeted gas sensing, gas separation, and related applications.

## Graphical abstract

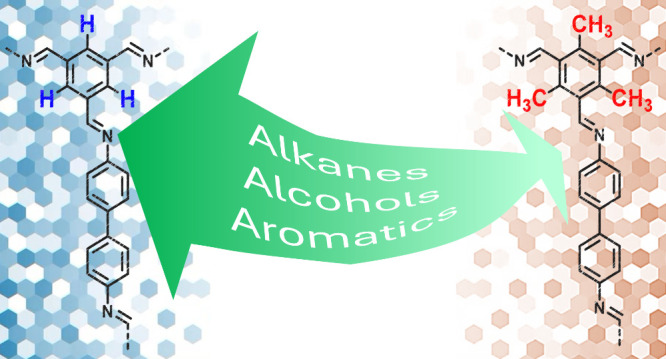

## 1 Introduction

Interactions between gases and materials play an important role in a variety of industrial processes, ranging from microchip fabrication to fuel production and exhaust gas cleaning systems.^1–3^ For these applications, porous nanomaterials derived from organic building blocks offer unique advantages. Their preparation allows tuning of the cavity properties through the use of functionalized building blocks or via postsynthetic functionalization schemes.^4^ A key strength of nanoporous organic materials is their ability to enable selectivity not only based on shape and size, but also through specific interactions between target analytes and the functional groups of the material, i.e., the adsorbent.^5^ As the material properties can also be tuned by introducing specific functionalities, it is crucial to understand, and ultimately be able to predict, how these functional groups impact the material properties. Within this context, the adsorption behavior toward volatile organic compounds (VOCs) is of particular interest.^6,7^ Therefore, mapping the interactions between porous nanomaterials and VOCs and their influence on the material properties is key in designing new adsorbents with targeted selectivity.

Covalent organic frameworks (COFs) are a class of nanoporous crystalline materials constructed from organic building blocks interconnected by strong covalent bonds. These frameworks offer tunable surface properties, and additional features like high chemical and thermal stability due to their strong π-conjugated backbone structure,^8^ making them highly suitable for gas adsorption applications.^9–13^ COFs also possess large surface areas (1000–5000 m^2^/g), enabling them to not only have high adsorption capacities, but also adjustable porosity.^14^ A broad array of pre- and postsynthetic COF modifications is available, including amination, hydroxylation and methylation.^15–19^ Examples of COF-gas interactions that result from COF modification include π–π stacking and van der Waals (vdW) interactions. Despite the growing interest in COF functionalities, their effect on gas interactions is not fully understood.^20^ Several factors play a crucial role in this lack of understanding, such as the complex pore environment and limited knowledge on the interactions occurring inside and outside of the pores. Furthermore, the use of advanced techniques beyond standard Brunauer-Emmet-Teller (BET) surface area and desorption analyses using gases like N_2_ and CO_2_ is still in its infancy.^14^ Material development in the field of COFs, as well as their exploration and implementation in targeted adsorption-based applications, can further benefit from more in-depth gas adsorption studies that allow inclusion of a large variety of gases under controlled conditions and enable acquisition of the involved thermodynamic properties.

Out of the various COF functionalities that can be targeted for gas adsorption, methyl groups are interesting because they improve several important material properties. Methyl groups have, for example, been shown to improve COF stability, increase their BET surface area, and allow for tunable UV absorption.^21^ By comparing the properties of methylated vs nonmethylated COFs, we aim to better understand their gas adsorption properties and ultimately aim to optimize and predict their interactions with VOCs, moving beyond the current trial-and-error approach in material development.

A series of imine-based COFs was previously synthesized in our laboratory, which are among the most widely studied framework materials,^22,23^ including methylated Me_3_TFB-PA and Me_3_TFB-BD, as well as nonmethylated TFB-PA and TFB-BD (Scheme S1).^21^ Within this series, the methylated COFs demonstrated significant improvements in structural and optical properties, particularly after exposure to hydrochloric acid vapor. While these improvements indicate their potential for various applications, their performance in adsorption-based processes such as gas sensing and separation, remains largely unexplored, aside from preliminary adsorption tests using hydrochloric acid vapor and the standard gases N_2_ and CO_2_.^21^

Inverse Gas Chromatography (IGC) has proven to be a powerful tool for investigating the interactions between nanoporous materials and volatiles of interest, referred to as probes in this study.^24,22^ The principle behind IGC is that a material is packed into a column, allowing gas-phase probes to be injected onto the packed material. The resulting retention times (*t*_r_) of the probes provide a measure of interaction strength, which can be converted into adsorption parameters like enthalpies, partition coefficients and selectivities. Over the last two decades, a variety of nanomaterials have been subjected to IGC analyses to gain insight into gas-material interactions. Examples of such nanomaterials include Metal–Organic Frameworks (MOFs)^24^ like Prussian Blue Analogues (PBAs),^25,26^ and COFs.^10,27^ Some studies report on the analysis of thermodynamic properties of COFs, including those of poly- and single crystalline 2D COFs, displaying excellent performances for their use in the separation of hydrocarbons. While a substantial number of publications describe the use of COFs in separation-based applications, there is only a limited number of studies that evaluate the influence of COF functional groups on the gas adsorption properties for additional applications, including gas sensing and gas removal. Often, in IGC analysis, COF-based stationary phases are prepared by coating capillary columns, typically 20–30 m in length, using COF suspensions.^10,17^ Recently, we developed a solvent-free packing method for a MOF, enabling the direct analysis of its adsorption properties without the need of a column coating process.^25^ Building on this method, in this work imine-based COFs were investigated, with a focus on the effect of methyl moieties on gas adsorption properties toward various VOCs using IGC. Nonmethylated TFB-BD and its methylated equivalent Me_3_TFB-BD were selected as a pair to evaluate the effects of methylation. The interactions of both COFs with specific target probes, toluene and heptane (*n*C_7_), were compared by acquiring their enthalpies. To further investigate the influence of methyl groups on VOC adsorption properties, a broader range of hydrocarbons was screened, including alkanes, alcohols and alkylbenzenes. To expand the scope and obtain further insight into the effect of methyl group density on the gas adsorption properties, an additional methylated COF, Me_3_TFB-PA, was included in this study. The adsorption capacities for all three COFs were then experimentally determined by injecting increased amounts of toluene and *n*C_7_. Finally, the selectivity of these COFs was evaluated using a broad set of injected probes.

## 2 Experimental Section

### 2.1 Materials

1,4-Phenylenediamine (>98% (GC)­(T)), 2,4,6-trimethylbenzene-1,3,5-tricarbaldehyde (95%) and benzene-1,3,5-tricarbaldehyde (96%) were purchased from TCI Europe N.V (Zwijndrecht, Belgium). Benzidine (98%) was purchased from Abcr GmbH (Karlsruhe, Germany) Mesitylene (99%) was purchased from Fisher Scientific (Waltham, MA, USA) and 1,4-dioxane (99%) was purchased from Acros Organics (Geel, Belgium). Methane (99%) and *n*-propane (99%) were purchased from SOL (Monza, Italy), ethane (99%) from Messer (Bad Soden, Germany), *n*-butane (actually, 80/20 *n*-butane/propane) from Campingaz (Saint-Genis-Laval, France). For *n*-butane, separation of both gases in this mixture was observed, with *n*-propane matching the retention time of its pure component. *N*-pentane (99%) from Merck (GaA (Darmstadt, Germany), *n*-hexane (99%) and toluene (99%) from Honeywell (Charlotte, NC, USA). *N*-heptane (99%), *n*-octane (98%), and 2-methylpropan-2-ol (99%) were purchased from Sigma-Aldrich (St. Louis, MO, USA). Ethylbenzene (99.8%) was purchased from Thermo Scientific (Waltham, MA, USA). All chemicals were used as received.

### 2.2 COF Synthesis and Characterization

All COFs were synthesized following a protocol previously published by Dautzenberg et al.^21^ In short, in a condensation reaction, either 2,4,6-trimethylbenzene-1,3,5-tricarbaldehyde (Me_3_TFB) or benzene-1,3,5-tricarbaldehyde (TFB) was reacted with 1,4-phenylenediamine (PA) or benzidine (BD) for 3 days in the presence of acetic acid under atmospheric pressure at 70 °C. The resulting COF powders underwent washing with DMF (2×), ethanol (1×) and acetone (1×), to prevent pore collapse, followed by overnight drying at 120 °C. A more detailed description of the experimental procedure is provided in the SI.

Fourier transformation infrared (FT-IR) measurements were carried out using a Bruker Tensor II (Billerica, MA, USA) with an attenuated total reflectance (ATR) crystal in transmission mode. A background spectrum was acquired prior to each measurement (*n* = 16). Next, a spatula tip of the respective COF powder sample was placed on the ATR crystal and 32 spectra were taken for each sample at a resolution of 4 cm^–1^.

For powder X-ray diffraction (PXRD) analysis, small amounts of each COF were placed onto a (553)-silicon wafer. The diffractograms were then obtained using a Panalytical Empyrean diffractometer in Bragg–Brentano geometry using CuKα radiation in a sealed LFF tube (45 kV, 40 mA) and a PIXcel3D 1 × 1 detector. Scan range (continuous) was between 2°< 2θ < 45° with a step size of 0.013°. The pore sizes and intersheet distances were determined through fitting the pore size (*a*, in meters) for the Miller indices.

The thermal stability was characterized via thermal gravimetric analysis (TGA) on an STA 6000 instrument (PerkinElmer, Waltham, MA, USA) under a continuous N_2_ stream, covering a temperature range of 298–773 K.

Nitrogen adsorption–desorption measurements were recorded at 77.350 K on a MicroActive for Tristar II Plus 2.01 after degassing the COF powders at 120 °C overnight. BET surface areas were determined from the adsorption data using the Rouquerol criteria: the upper bound of the linear regime was set at the maximum of *n*(1 – *p*/*p*°) vs *p*/*p*° and the lower bound was chosen so that the difference between ( $1/(\sqrt{C+1)}$ and the *p*/*p*° at *n*_m_ was <10% (or minimized). Pore-size distributions were derived with the “N2–Cylindrical Pores–Oxide Surface” model, using a selected regularization parameter to balance fit quality and smoothness.^28^

### 2.3 IGC Equipment and Column Packing

Glass tubes measuring 78 mm × 3 mm (length × internal diameter) were used to assemble IGC columns. One end of the tube was sealed with dimethyldichlorosilane (DMCS)-treated glass wool, kept in place with a stainless-steel triangular clamp, to prevent any material from leaving the column. After sealing, the tube was connected to a vacuum pump. A small glass funnel was securely positioned at the top opening of the vertically oriented tube to insert approximately 0.1 g of COF inside the tube (Figure 1a). After removal of the funnel, the COF powder (Figure 1b) was then pushed down with a wooden stick matching the tube internal diameter, to give a homogeneous packing (Figure 1b). After packing, the other side of the tube was sealed off with additional glass wool, before installing it into a modified Agilent 6850 series II Gas Chromatograph (Amstelveen, The Netherlands) equipped with an FID detector. Subsequently, the column underwent overnight N_2_ conditioning to eliminate any residual moisture and stabilize the detector.

**Figure 1:**
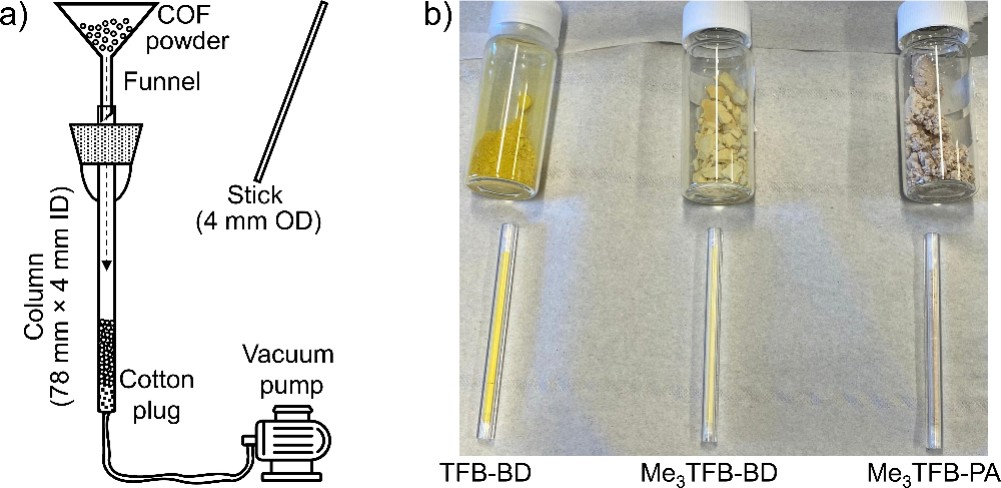
(a) Schematic illustration of the COF-packing method (not to scale). A stick with the same diameter as the column is used to push the COF particles downward, while the applied vacuum ensures uniform packing. (b) Photograph of vials with the as-synthesized COF powders (top) and the packed columns (bottom) utilized within this work.

### 2.4 IGC Parameters

Enthalpy measurements were performed by injecting 0.1 μL probe volumes at temperatures between 150 and 180 °C at steps of 10 °C. A 10-min waiting period between temperature adjustments was applied to allow the system to stabilize. During each series of injections at a specific temperature, methane (C_1_) was injected as a *t*_0_ void-time marker (also known as *t*_M_ in some studies). By measuring the retention times (*t*_r_) at different temperatures, the enthalpy can be derived from the general Arrhenius equation:

$$k=Ae^{-\Delta H_{\mathrm{ads}}/RT}$$ 1

where *k* is the retention factor calculated as the retention time (*t*_r_) corrected for the void time (*t*_0_) *t*_r_ – *t*_0_/*t*_0_, *A* the pre-exponential factor, *R* the ideal gas constant (8.314 J mol^–1^·K^–1^) and *T* the temperature in Kelvin. The slope, determined by plotting ln *k* vs 1/*T*, of the Arrhenius plot can be utilized to calculate Δ*H*_ads_:

$$-\Delta H_{\mathrm{ads}}=\mathrm{slope}\times R$$ 2

The IGC experiments for obtaining the partition coefficients (*K*) were performed at a single temperature of 150 °C. The inlet pressures of the packed COF columns were adjusted to achieve a flow rate of 1.5 mL/min. Specifically, pressures of 165, 76, and 41 kPa were applied to the Me_3_TFB-PA, TFB-BD, and Me_3_TFB-BD columns, respectively. Peak asymmetries due to void volumes in the packed bed were occasionally observed. In such cases the column was repacked. Prior to each series of experiments, methane (C_1_) was injected as a *t*_0_ void-time marker, and the instrument was heated to the desired temperature for 30 min to stabilize the detector. Probe volumes for attaining the partition coefficients were either 0.1 μL of liquid or 25 μL of gas.

The partition coefficients were calculated using eq 3 to normalize the column surface area and COF mass:

$$K=\frac{V_{N}}{\sigma \times m_{s}}$$ 3

Herein, *K* is the partition coefficient related to the probe concentration in the mobile and stationary phase in cm^3^·m^–2^, *V*_N_ the net retention volume (cm^3^), σ the N_2_-based BET surface area of the column material in m^2^/g, and *m*_s_ the mass of the utilized COF powder inside the column in g.

Adsorption capacity measurements involved injecting increasing probe volumes from 0.1 μL to 1 μL at various split ratios to oversaturate the COF column material. Experiments were performed at *T* = 150 °C. The adsorption isotherms were calculated from the obtained retention volumes using eq 4:

$$n_{\mathrm{ad}}=\frac{1}{m_{s}}\cdot \int \frac{V_{N}}{RT}dP$$ 4

Here, *n*_ad_ represents the adsorbed amount per g of COF material in mol/g; *R* the ideal gas constant and *T* the experimental temperature in K; and *m*_s_ the mass of the respective COF powder inside the column in g. To account for any differences in surface area, all *n*_ad_ values were divided by the respective surface area of the COFs, resulting in a mol/m^2^ unit.

Kovats Retention Indices for each probe per COF were calculated using eq 5.^29^ The *K* values of all probes were organized in such a way that they elute between the alkanes. Retention index differences were then determined following the approach of McReynolds and Rohrschneider,^29^ by setting the retention index for each probe on TFB-BD to 0 (i.e., subtracting its own index value), and calculating the differences for the other COFs as Index­(Me_3_TFB-BD)–Index­(TFB-BD) and Index­(Me_3_TFB-PA)–Index­(TFB-BD), respectively.

$$I_{i}=100[n+\frac{\log(t_{i}-t_{0})-\log(t_{n}-t_{0})}{\log(t_{n+1}-t_{0})-\log((t_{n}-t_{0})}]$$ 5

Herein, *I*_*i*_ denotes the Kovats retention index of peak *i*, *n* the number of carbon atoms of an *n*-alkane peak eluting before peak *i*, and *t*_*i*_ represents the retention time of compound *i* in minutes.

## 3 Results and Discussion

### 3.1 COF Powder Characterization

Prior to their packing in the IGC columns, the synthesized COFs were first characterized using Fourier-transform infrared (FT-IR) spectroscopy, thermogravimetric analysis (TGA) and gas adsorption measurements, including Brunauer–Emmett–Teller (BET) surface area, pore size analysis, and powder X-ray diffractometry (PXRD). The FT-IR and TGA data can be found in Figures S1 and S2 and are in line with previously reported data,^21^ confirming the successful synthesis of the COFs. In short, the FT-IR bands at 1623 and 1689 cm^–1^ (Figure S1) point to the presence of an imine moiety and residual carbonyl groups, respectively. Furthermore, the band near 2865 cm^–1^ of the FTIR spectrum of the nonmethylated COF is characteristic for the C–H stretching vibration of N=C–H moieties that are part of an aromatic system, like the one of reference compound N,1-diphenylmethanimine.^30^ The bands in this part of the FTIR spectra of the two methylated COFs are broader and can be assigned to saturated C–H stretching vibrations, pointing to the presence of CH_3_ groups. All COFs show bands just above 3000 cm^–1^, which can be assigned to the unsaturated C–H stretching vibrations of the aromatic rings. TGA analyses shows a high thermal stability for all three materials up to at least 360 °C (Figure S2). The BET analysis data (Figure S3) demonstrated surface areas of 1426 m^2^/g for TFB-BD, 1910 m^2^/g for Me_3_TFB-BD, and 2218 m^2^/g for Me_3_TFB-PA, again in line with literature.^21^ Calculated average pore sizes (Figure S4) were found to be 1.9 nm for both TFB-BD and Me_3_TFB-PA and 2.5 nm for Me_3_TFB-BD, which is in line with with previously calculated values using non-local density functional theory (NLDFT) analysis.^21^ Realizing that small-volume functional groups have a minimal effect on the pore size of COFs,^31^ the BET and pore size data suggests that TFB-BD is partly collapsed.^32^ PXRD patterns of the as-synthesized powder (Figure S5) show that crystallinity of the COFs is similar to that reported previously, with diffraction peaks at 4.9, 8.2, 9.5, 12.5, 16.3 and 24.2° for Me_3_TFB-PA, corresponding to the reflection of the (100), (110), (200), (210), (220) and (001) planes, respectively.^21^ A slightly different pattern is found for Me_3_TFB-BD, with peaks at 3.6, 6.1, 7.0, 9.3, 12.1, 12.6, 15.2 and 23.9°, corresponding to the reflection of the (100), (110), (200), (210), (220), (310), (320) and (001) planes. TFB-BD shows three overlapping peaks with Me_3_TFB-BD at 3,6, 6.1 and 7.0°. The pore sizes calculated from the PXRD data are 2.1, 2.8, and 2.9 nm in diameter for Me_3_TFB-PA, TFB-BD and Me_3_TFB-BD, respectively. These slightly higher values, compared to those from pore size distribution data (Figure S4) and prior domain size analysis, can be rationalized by the calculation method, which accounts for the thickness of the pore walls. When comparing the results of the COF materials that have undergone packing and adsorption experiments to the native powders, the data suggests that the crystallinity has remained consistent upon packing and multiple adsorption cycles performed with IGC, indicating high stability under these conditions. No observable changes in material properties or adsorption performance were detected following adsorption experiments, as shown via PXRD analysis and consistent retention time data for the *t*_0_ void marker (Figure S6).

### 3.2 IGC Analysis of Methylated and Nonmethylated TFB-BD

#### 3.2.1 Enthalpies of Toluene and Heptane Adsorption

After packing Me_3_TFB-BD and its nonmethylated equivalent TFB-BD (Figure 2a for structural information) in IGC columns, an initial IGC screening of their adsorption properties was performed. This screening aimed to determine the difference in enthalpy of adsorption (Δ*H*_ads_) for aliphatic and aromatic hydrocarbons. Toluene and *n*C_7_ (heptane) were selected as probes for this purpose and were introduced into the packed IGC columns at multiple temperatures in the temperature range of 150–180 °C.

**Figure 2:**
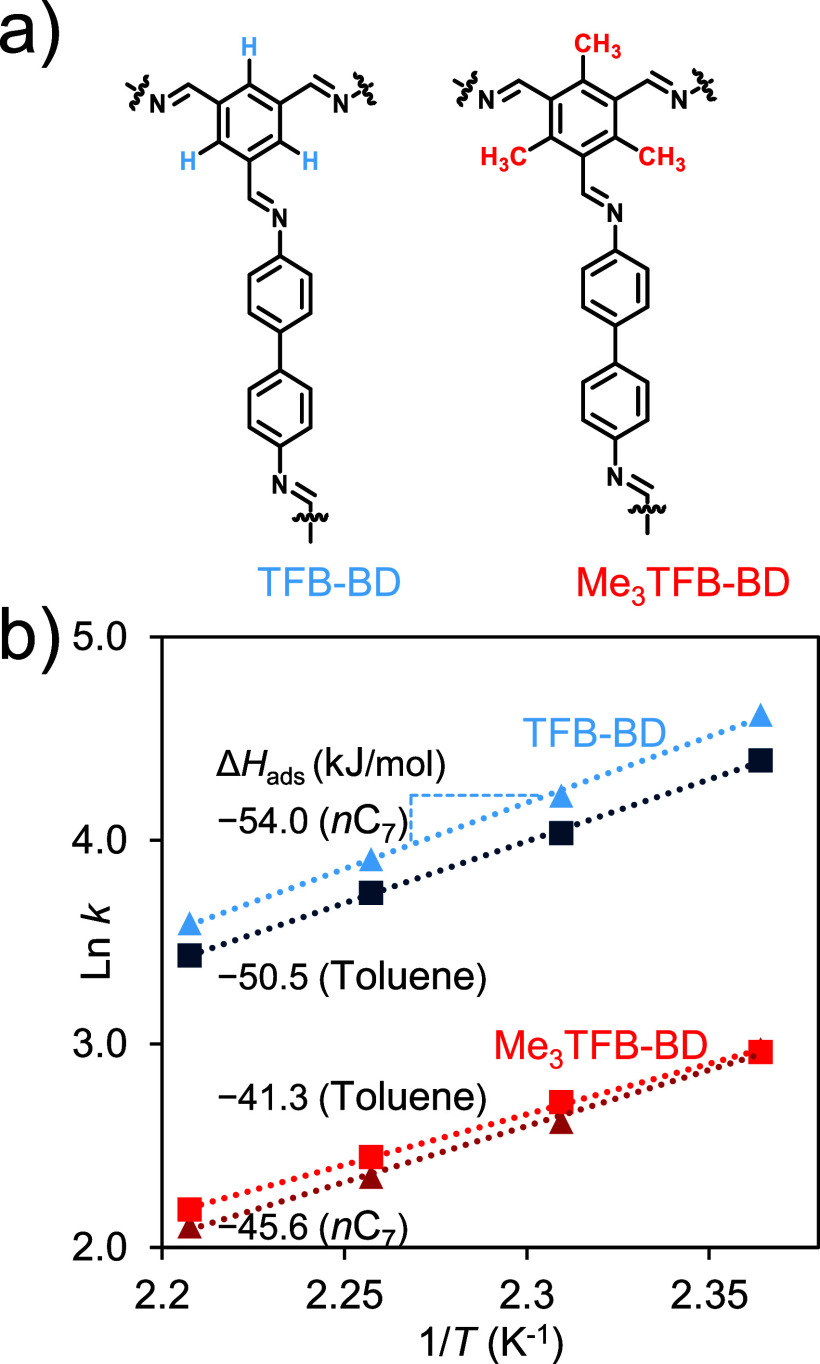
(a) Structural differences between TFB-BD and Me_3_-TFB-BD COFs as highlighted in blue and red, respectively, (b) Arrhenius plot of the Me_3_TFB-BD and TFB-BD for two different probes.

Figure 2b presents the Arrhenius plot derived from these measurements, along with the calculated enthalpy values for the injected probes toluene and *n*C_7_ onto both COFs. The enthalpy values were calculated according to eq 2. For Me_3_TFB-BD, the Δ*H*_ads_ values for toluene and *n*C_7_ are −41.3 and −45.6 kJ/mol, respectively. Contrary, for TFB-BD, the Δ*H*_ads_ values for toluene and *n*C_7_ are −50.5 and–54.0 kJ/mol. For TFB-BD, *n*C_7_ elutes later than toluene at all measured temperatures (150–180 °C). However, for Me_3_TFB-BD, a reversal of the elution order is seen with toluene eluting later than *n*C_7_. Overall, for these COFs, the enthalpy data suggests that π–π interactions do not play a major role for both probes. In contrast, the imine bonds appear to play a more prominent role in the interactions with the probes. These bonds may induce (temporary) polarization of the probes, leading to dipole–dipole interactions. Additionally, dispersive forces like vdW interactions with *n*C_7_, and to a lesser extent with toluene, may play a role in this enhanced interaction.

Realizing that the type of COF utilization in this study (i.e., the packed powder form in a 7.8 cm column) may have influenced the thermodynamic parameters, we compared our data to those in previous studies^10,33^ wherein COFs were used as a coated stationary phase of a longer, 20 m IGC column. The adsorption enthalpies obtained from our experiments align well with those of hydrocarbon interactions in these other studies. For *n*C_7_, for example, Dichtel and co-workers reported enthalpy values for polycrystalline (−58.6 kJ/mol) and single crystalline (−72.6 kJ/mol) imine-based COFs.^10^ This difference between poly- and single crystalline COFs was attributed to surface inhomogeneities and pore-edge irregularities, which created additional adsorption sites in the COF pores and led to stronger interactions than those observed in single crystals. In another study,^33^ the data set of Dichtel and co-workers was analyzed using a more advanced model, leading to enthalpy values of −44.7 to −69.7 kJ/mol for the *n*C_5_–*n*C_8_ range, values similar to those observed in the current study.

#### 3.2.2 Probing π–π Interactions

To further assess the influence of probe aromaticity and the role of imine bonds on the retention behavior of both COFs, additional aromatic, cyclic and aliphatic alkanes were injected and studied in more detail. These probes included benzene, styrene, cyclohexane and methylcyclohexane. As shown in Figure 3, the partition coefficients (*K*), calculated using eq 3, for TFB-BD vary between 0.1 and 2.0 cm^3^/m^2^, whereas Me_3_TFB-BD exhibits lower values (0.03–0.5 cm^3^/m^2^). For both COFs, benzene and toluene have lower *K* values compared to their aliphatic and cyclic counterparts, that is versus *n*C_6_ and *n*C_7_ and cyclohexane and methylcyclohexane, respectively.

**Figure 3:**
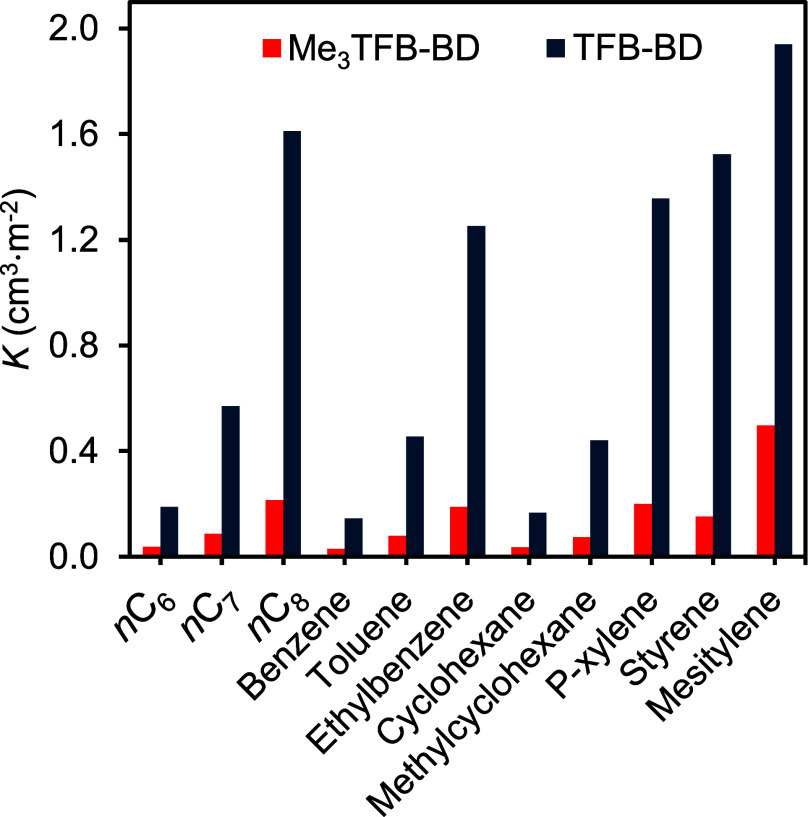
Partition coefficient (*K*) values for aliphatic and aromatic hydrocarbon probes for Me_3_TFB-BD (red) and TFB-BD (blue). Experimental *T* = 150 °C.

Notably, the highest *K* value is observed for mesitylene, with values of approximately 2.0 cm^3^/m^2^ for TFB-BD and 0.4 cm^3^/m^2^ for Me_3_TFB-BD. In line with the enthalpy data, the *K* results suggest that there is a stronger interaction of all probes with TFB-BD compared to Me_3_TFB-BD, likely due to the absence of methyl groups surrounding the imine bond. Similarly, aromatic probes show lower *K* values than their linear and cyclic aliphatic counterparts. For instance, hexane, benzene and cyclohexane have comparable *K* values, supporting the conclusion that π–π interactions only play a minimal role in the COF-probe interactions.

Instead, the data suggest that dispersive contributions play a more prominent role, which becomes particularly clear from the *n*-alkane probe series, as it displays an increasing *K* value with increasing chain length. Probe polarizability also appears to influence the interaction strength, possibly due to dipole–dipole interactions with the imine bonds. For example, styrene has a higher *K* value than ethylbenzene and *p*-xylene for both COFs. Additionally, a combination of steric and electronic effects could contribute to the different interactions.

#### 3.2.3 Partition Coefficients of Alkyl Series

So far, only π–π and dispersive interactions, like vdW, have been probed for both TFB-BD and Me_3_TFB-BD. To further validate the importance of imine bonds in COF-probe interactions and investigate any additional interactions, a broader range of hydrocarbons was studied, including a series of alkanes (*n*C_2_ to *n*C_8_), alcohols (C_1_–OH to *n*C_6_–OH) and alkylbenzenes (benzene, toluene and ethylbenzene). Figure 4 illustrates the *K* values of these probes injected on TFB-BD (Figure 4a) and Me_3_TFB-BD (Figure 4b), from which it becomes clear that TFB-BD also exhibits higher *K* values compared to Me_3_TFB-BD for the injected probe series.

**Figure 4:**
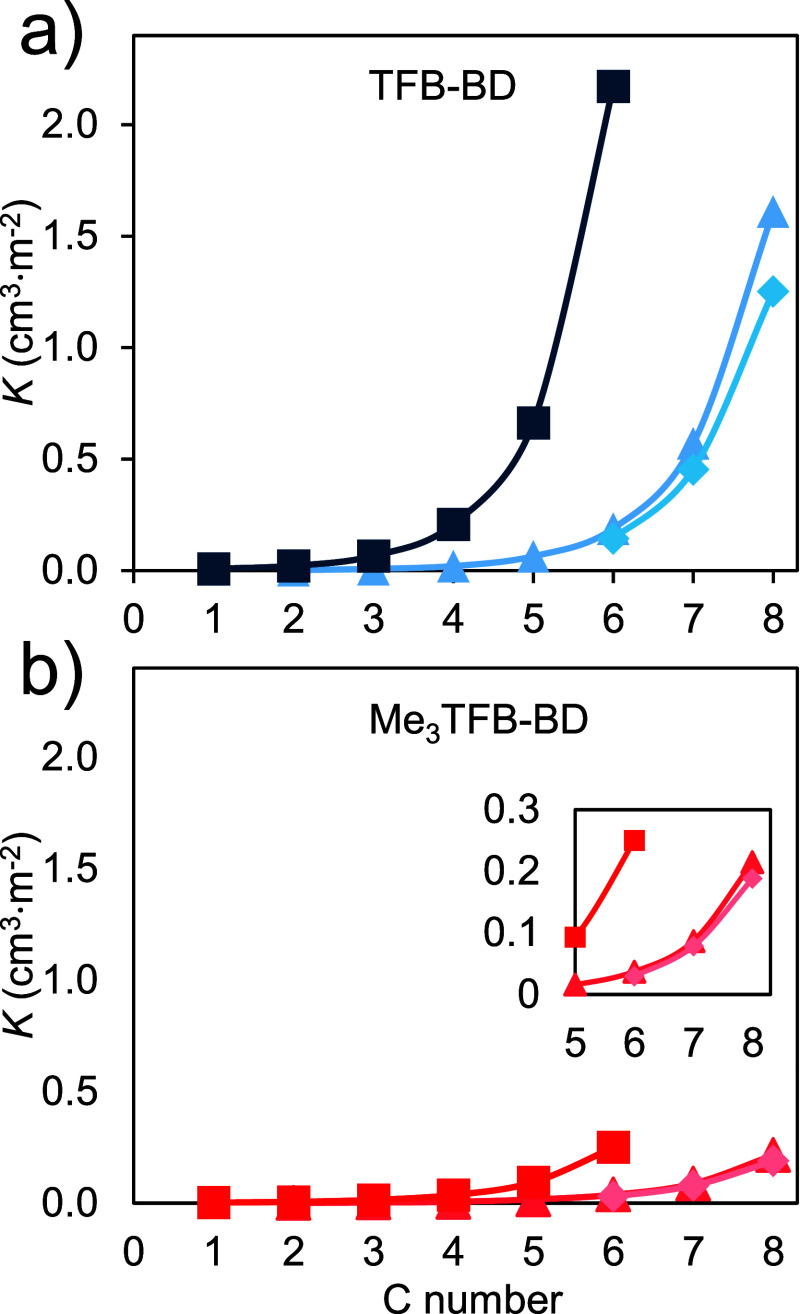
*K* values for (a) TFB-BD and (b) Me_3_TFB-BD as function of the number of carbon atoms, wherein the probe values correspond to alkanes (◆), alcohols (■), and alkylbenzenes (▲). Experimental *T* = 150 °C.

In their interactions with probes, the differences between these COFs are more pronounced for alkanes with larger alkyl chain lengths, such as *n*C_8_, where TFB-BD displays a *K* value of 1.6 compared to 0.2 for Me_3_TFB-BD. This trend further supports the role of the alkyl chain length in increasing the dispersive interactions, as the K values increase with increasing alkyl chain length. Interestingly, the alcohol probes display much stronger retention on both COFs compared to their aliphatic and aromatic counterparts. For example, for TFB-BD, hexanol (*n*C_6_–OH) shows a partition coefficient approximately 11 times larger than its aliphatic (*n*C_6_) and aromatic (benzene) counterparts (∼2.2 vs ∼0.2). For Me_3_TFB-BD, the increase between *n*C_6_–OH, *n*C_6_ and benzene is approximately a factor of 6 (∼0.25 vs ∼0.04). These enhanced retentions can be rationalized by the presence of the polar OH group, leading to dipole–dipole interactions between the alcohol and imine moieties. The presence of methyl groups near the imine bonds of Me_3_TFB-BD may explain a reduction of this effect. These findings support the hypothesis that the imine bonds play a key role in COF-probe interactions.

#### 3.2.4 Influence of Methyl Group Density and COF Structure

The consistently higher *K* values for TFB-BD compared to its methylated equivalent, Me_3_TFB-BD, suggests reduced π–π interaction availability, a dispersive contribution of the alkyl chain length, and the key role of imine bonds in the interactions. To further validate these results, a different yet structurally related methylated COF, Me_3_TFB-PA,^21^ was investigated. This additional framework possesses a higher methyl-to-carbon ratio, as it is built using a smaller diamine linker. It would have been interesting to also include its nonmethylated equivalent (TFB-PA) in this study, but the poor stability of this COF under atmospheric conditions led to its exclusion in this investigation. As shown in Figure S7, the *K* values for the tested probes decreased further for Me_3_TFB-PA (Figure S7a), supporting the line of thought that an increased methyl-to-carbon ratio reduces the interaction strength. The enthalpy values for Me_3_TFB-PA also followed this trend (Figure S7b), with lower values observed for *n*C_7_ (−34.4 kJ/mol) and toluene (−34.6 kJ/mol). After verifying that these enthalpy value**s** were lower for Me_3_TFB-PA compared to the other two COFs, all three frameworks were further compared, by measuring their adsorption capacity and selectivities toward a broad number of probe analytes.

#### 3.2.5 Adsorption Capacity

All experiments described so far were performed at concentrations within the linear adsorption regime, where the relationship between concentration and adsorbed amount is still proportional. To further quantify the adsorption behavior of the three selected COFs outside of this linear regime, their adsorption capacities for toluene and *n*C_7_ were obtained. The results are shown in Figure 5, wherein the adsorbed amounts are plotted as a function of the partial pressure, following the Peak Maximum (PM) method according to eq 4.^25^ In this method, the obtained *t*_r_ values corresponding to the maximum peak intensity of each injected probe concentration are used to calculate an adsorption isotherm. A shift in *t*_r_ toward lower retention times at higher injected amounts indicates that saturation of the column material starts to occur, resulting in a flattened adsorption isotherm around 0.3 kPa for TFB-BD and 0.8 kPa for Me_3_TFB-BD.

**Figure 5:**
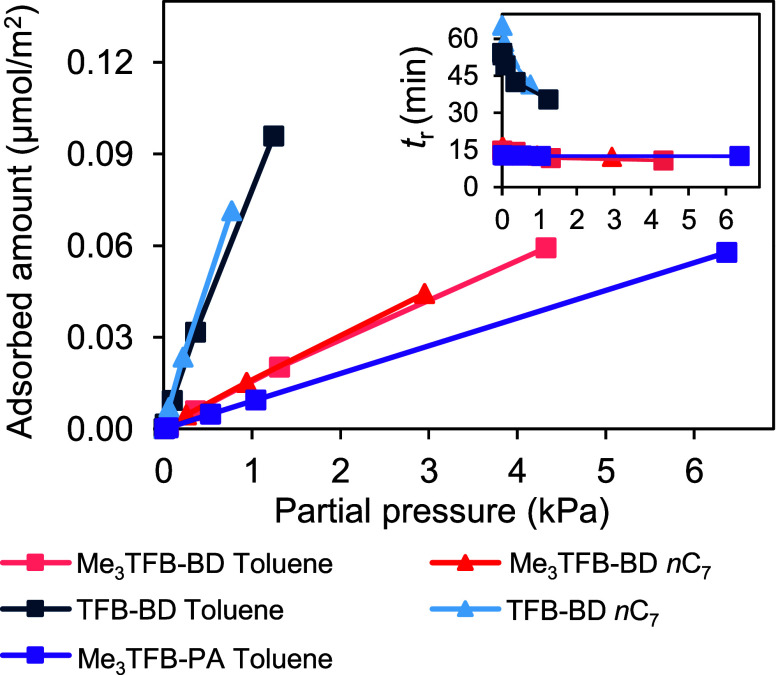
Adsorption capacities of Me_3_TFB-BD (red), TFB-BD (blue) and Me_3_TFB-PA (purple) for toluene and *n*C_7_. The inset displays the respective retention times (*t*_r_) for each injection at increasing amounts. Experiments were performed at *T* = 150 °C.

Figure 5 shows that for Me_3_TFB-BD, the adsorbed amount for *n*C_7_ is higher than that of toluene across all partial pressures. A similar trend is observed for TFB-BD, where *n*C_7_ and toluene display the highest adsorbed amounts, reaching an adsorption of ∼0.07 μmol/m^2^ at ∼0.75 kPa. The use of μmol/m^2^ is useful for comparing different COFs and probes due to normalization of the surface area with respect to the adsorbed amounts. However, to facilitate comparison with literature data, the values obtained in this study have also been converted to mg/g. These converted values are listed in Table 1, together with the adsorbed amounts in μmol/m^2^. The converted amounts in mg/g are comparable to reported values in literature. For instance, values for toluene are comparable to a wide range of reported values for some porous materials like MOFs (e.g., 6–397 mg/g).^34^ While data on toluene adsorption onto COFs in powder form is scarce, there are reported values for COF-based membranes.^35,36^ Those values are higher than reported in this study, as our experiments were not aimed at reaching saturation. It is also clear that, for both probes, the adsorption on the nonmethylated COFs is faster compared to the methylated COFs. This is in line with differences in the probe-imine interaction strength discussed above. The injections at lower concentrations were performed in duplicate to validate the reproducibility, along with the time of the void-marker (methane). The results of these injections are shown in Figure S6 and indicate that the retention times of both *n*C_7_ and toluene remained similar across multiple injections. In addition, the *t*_0_ values were also comparable throughout the measurements.

**Table 1:** Overview of the Adsorbed Amounts (in Two Units) of Toluene and Heptane onto the Studied COFs, Obtained at *T* = 150 °C upon Increasing Injection Amounts

COF	probe	adsorbed amount(μmol/m^2^)	adsorbed amount(mg/g)
TFB-BD	toluene	0.1	12.6
TFB-BD	*n*C_7_	0.07	10.2
Me_3_TFB-BD	toluene	0.06	10.4
Me_3_TFB-BD	*n*C_7_	0.04	8.4
Me_3_TFB-PA	toluene	0.06	12.8

The inset of Figure 5 further highlights the observed *t*_r_ shifts for all three COFs, where TFB-BD and Me_3_TFB-BD do not reach full saturation within the tested concentration range. In contrast, Me_3_TFB-PA demonstrates a linear response for all injected amounts of toluene, as indicated by the absence of a *t*_r_ shift in the inset of Figure 5. A similar trend is observed for *n*C_7_, indicating no oversaturation (data not shown), for Me_3_TFB-PA within the studied range. The magnitude of the *t*_r_ shift varies between 30% (Me_3_TFB-BD) and 40% (TFB-BD) for the minimum and maximum injected amount, respectively.

The larger *t*_r_ shift for TFB-BD indicates stronger interactions and a higher uptake of both probes at low vapor concentrations compared to the methylated COFs. However, despite its stronger initial adsorption, TFB-BD displays a lower overall adsorption capacity and rapid saturation. The strong adsorption behavior at low vapor concentrations, combined with a limited adsorption capacity, makes this COF an attractive material for sensing purposes, where rapid and sensitive detection plays an important role. Conversely, Me_3_TFB-BD shows a higher normalized adsorption capacity, likely due to its larger pore size and reduced interaction strength, making it more suitable as an adsorbent. Next to these differences in adsorption capacity, the ability of these COFs to selectively interact with specific hydrocarbons is an important factor to consider to for their practical applicability.

#### 3.2.6 Hexane Isomer Selectivity

To assess the applicability of all three COFs for selective hydrocarbon adsorption purposes, four *n*C_6_ isomers were injected along with *n*C_6_. These isomers are relevant in various fields,^37^ including fuel production and consumption, as their separation is required for efficient removal of the formed byproducts. However, the separation is challenging due to their similar kinetic diameter and boiling point.^38,39^ The *n*C_6_ separation results for all three COFs are presented in Figure 6, wherein the selectivity α was calculated as the ratio of the net retention time of each probe to that of *n*C_6_. Lower α values indicate a higher difference relative to *n*C_6_, meaning less retention. The results show that α values increase with the bulkiness, vapor pressure and boiling point of the isomeric probes. For instance, within this COF series, 3-methylpentane (3MP) shows the highest α values (0.81–0.88), while 2,2dimethylbutane (22DMB) shows the lowest ones (0.63–0.74). Notably, TFB-BD exhibits the lowest α across all probes ranging from 0.63 to 0.81, followed by Me_3_TFB-PA (0.69–0.85) and Me_3_TFB-BD (0.71–0.88). The kinetic diameter of all injected isomers is around 0.6 nm,^39^ more than 3-fold lower than all respective COF pore diameters, indicating that all probes can access the pores, thereby excluding processes like size-based exclusion. The α values closer to unity observed for Me_3_TFB-BD and Me_3_-TFB-PA suggest an increased relative interaction with the injected isomers. Furthermore, TFB-BD has the lowest α values for all isomers and therefore the highest selectivity, compared to *n*C_6_. Given the steric differences among the isomers, this result suggests that probe bulkiness has a limited impact on the interaction strength with these COFs.

**Figure 6:**
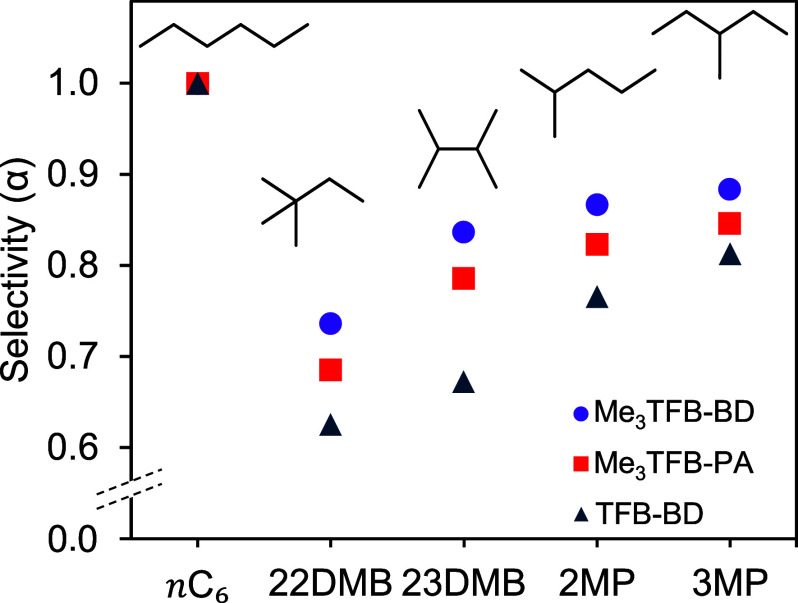
Hexane isomer selectivity (α) values for Me_3_TFB-PA (purple), Me_3_TFB-BD (red) and TFB-BD (blue), respectively. Probe abbreviations: 22DMB (2,2 dimethylbutane), 23DMB (2,3 dimethylbutane), 2MP (2-methylpentane) and 3MP (3-methylpentane). The selectivity is expressed versus *n*-hexane (*n*C_6_) and all experiments were performed at *T* = 150 °C.

#### 3.2.7 Separation Performance

Besides the selectivity toward hexane isomers, the efficiency of COFs in selective hydrocarbon adsorption is an important consideration. After looking into the selectivity of all three COFs toward the *n*C_6_ isomers, their selectivity for a broader range of probes was evaluated, providing more insights into their use in applications closer toward industry, such as gas separation and sensing. The selectivity of the different COFs was assessed by expressing the retention information for each probe relative to the retention times of an alkane series. Traditionally, in IGC, the retention of alkanes is believed to be due to dispersive interactions only. By comparing the retention times of different probe molecules with those of the alkanes, specific interactions between probe and COF can hence be expressed in a quantitative manner. A straightforward way to do so is by using Kovats retention indices. In the Kovats system, elution positions, or *K* values, of the probes are expressed relative to those of the alkanes. A high Kovats index of, e.g., an aromatic species, indicates a strong interaction of the aromatic probe with the respective COF powder. The Kovats system is also particularly useful for comparing results across different COFs. Setting the retention index of one COF to zero allows for a rapid comparison of the selectivity of the other materials to this reference (Figure 7).

**Figure 7:**
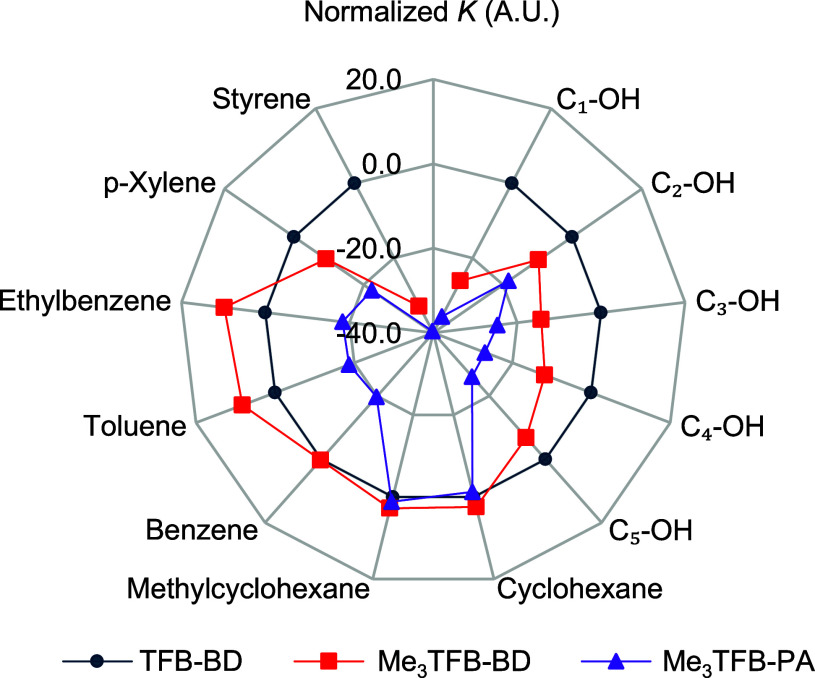
Calculated retention indices according to Kovats’ method for all probes injected onto Me_3_TFB-BD (red, squares), Me_3_TFB-PA (purple, triangles) and TFB-BD (blue, circles), respectively. The TFB-BD material was selected as the reference material. The lines extending toward the *x*-axis denote the Kovats index relative to that of TFB-BD. A line was drawn through each data series to serve as a visual guide for the reader.

Figure 7 shows that TFB-BD exhibits the highest retention index values for *p*-xylene, styrene and all tested alcohols compared to the other two methylated COFs (see Table S1 for all calculated values). For instance, the alcohol pentanol (C5-OH) exhibits a higher retention index on TFB-BD compared to Me_3_TFB-BD, indicating a stronger interaction of the hydroxyl functionalities with TFB-BD. This further confirms that, compared to the other two COFs, TFB-BD forms stronger dipole interactions, in particular with unsaturated carbon–carbon bonds and alcohols. In contrast, the retention indices for benzene, methylcyclohexane and cyclohexane are nearly identical for all three COFs, suggesting that the relative interaction differences are minimal with these probes. While the presence of methyl moieties may reduce overall adsorption strength, it can increase selectivity for specific probes. For example, Me_3_TFB-BD displays the highest values for toluene and ethylbenzene. This can be attributed to increased interactions between the methyl groups (and aromatic rings) present in toluene and ethylbenzene with methyl functionalities of the COFs. These specific interactions, while absent in the data previously presented in the Partition Coefficient data, reveal the usefulness of comparing materials using Kovats retention indices. This selective adsorption behavior highlights the potential of these COFs for gas sensing, hydrocarbon separation, and storage applications wherein targeted adsorption properties are crucial. While the differences are subtle, the consistent trends observed for TFB-BD indicate its promising use for incorporation into sensing devices.

## 4 Conclusions

The systematic IGC-based approach utilized within this study enabled us to map the gas adsorption properties of multiple methylated and nonmethylated COFs. The results indicate that TFB-BD, which lacks methyl groups, shows the strongest interactions with most of the injected hydrocarbons used in this study. This can be attributed to its increased imine bond accessibility compared to its methylated equivalent. In addition, given the π–π stacking between COF sheets, π–π interactions between the COF and aromatic probes are unlikely to play a role within the pore, suggesting that dispersive interactions like vdW interactions are more favored within these frameworks. This is supported by the effect of the chain length of the alkyl probe series on the *K* values. Furthermore, the strong interaction with alcohol probes, compared to the aliphatic and aromatic probes, with all three COFs, was rationalized by dipole–dipole interactions. Selectivity analysis highlighted that TFB-BD has the highest selectivity for hexane isomers, and in fact most probes in the series. Its adsorption capacity, however, is the lowest adsorption for toluene and *n*C_7_. In addition, the absence of methylation can also comprise stability, as demonstrated by the exclusion of TFB-PA from this study due to its increased risk of pore collapse. This trade-off between adsorption strength, selectivity, and stability offers valuable insights that can facilitate the tailoring of COFs for targeted gas adsorption. This study therefore not only advances our understanding of nanoporous COF behavior but also highlights their significant potential for implementation in gas detection, gas separation, and related fields.

## Supplementary Material



## Notes

COF structures, synthesis and related data, FT-IR, TGA, sorption isotherms and BET linearization plots, PXRD patterns, pore-size distributions, retention times, enthalpy values and partition coefficient data, and calculated Kovats retention indices (PDF)
