# Supplementary material for: Comparison of Gas Adsorption Properties in Methylated and Non-Methylated Imine-Linked Nanoporous Covalent Organic Frameworks
Source: ACS Appl Nano Mater. 2025 Sep 15;8(40):19145–53. doi: 10.1021/acsanm.5c02616 (PMC12519445; doi:10.1021/acsanm.5c02616)
Supplement: Supplementary file 1 [file an5c02616_si_001.pdf]

Supporting Information:

Comparison of Gas Adsorption Properties in Methylated and Non-methylated Imine-Linked Nanoporous Covalent Organic Frameworks

Stijn Paulusma[a], Thijmen A. van Voorthuizen[a], Hans-Gerd Janssen[a,b], Louis C. P. M. de Smet[a]*

[a]Laboratory of Organic Chemistry, Wageningen University Stippeneng 4, 6708 WE, Wageningen, The Netherlands

[b]Unilever Foods Innovation Centre — Hive, Bronland 14, 6708 WH, Wageningen, The Netherlands

*Email address: louis.desmet@wur.nl

Table of Contents

**Scheme S1.**	COF structures and synthesis	S2
**Figure S1.**	FT-IR spectra	S3
**Figure S2.**	TGA plots	S3
**Figure S3**.	Sorption isotherms and BET linearization plots	S4
**Figure S4**.	PXRD patterns	S4
**Figure S5**.	Pore-size distribution data	S5
**Figure S6**.	Retention time data	S5
**Figure S7**.	Enthalpy and partition coefficient data Me_3TFB-PA	S6
**Table S1.**	Calculated Kovats Retention Indices	S6

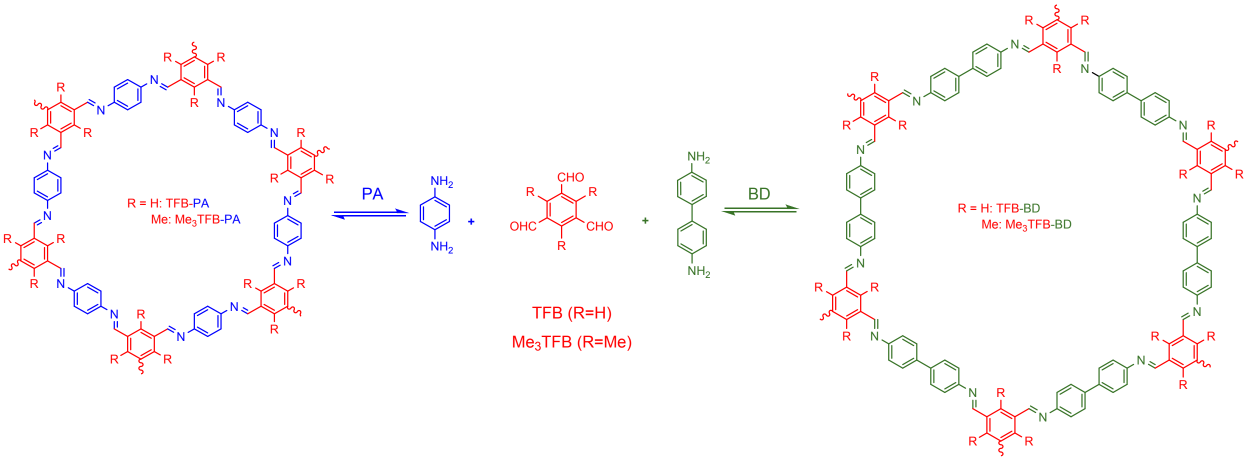

Scheme S1. Structures of selected covalent organic frameworks (COFs) that have been investigated for their adsorption properties using inverse gas chromatography (IGC). Benzene-1,3,5-tricarbaldehyde (TFB, R = H, in red) or 2,4,6-trimethylbenzene-1,3,5-tricarbaldehyde (Me$_3$TFB, R = Me, in red) reacts with either phenylenediamine (PA, blue) or benzidine (BD, green) to form imine-linked networks.

COF synthesis

Either 1,3,5-triformylbenzene (TFB, 250 mg, 1.542 mmol, 1 equivalents) or 2,4,6-trimethyl-1,3,5-triformylbenzene (Me$_3$TFB, 315 mg, 1.542 mmol, 1 equivalents) and either 1,4-phenylenediamine (PA, 250 mg, 2.313 mmol, 1.5 equivalents) of benzidine (BD, 426 mg, 2.313 mmol, 1.5 equivalents) were added to a 50 mL round-bottom flask and dissolved in 10.0 mL 1,4-dioxane:mesitylene 4:1 v/v while heating to 70 °C. Subsequently, 2.4 mL water and 3.6 mL glacial acetic acid were added, and a condenser was affixed to the set-up. The reaction mixture was stirred at 70 °C for 3 days under an ambient atmosphere and pressure.

The resulting precipitate was collected by Büchner filtration, and the flask was rinsed with DMF. The solids were then dispersed in 250 mL DMF and heated to 90 °C under stirring for 30 minutes, followed by Büchner filtration. This washing step was repeated with DMF, ethanol and acetone, respectively. Finally, the solids were dried overnight in an oven at 120 °C.

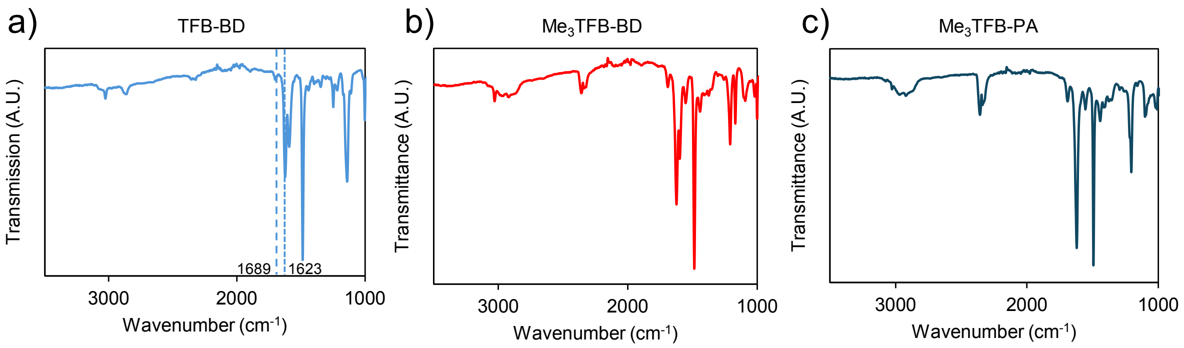

Figure S1. FT-IR spectra of a) TFB-BD, b) Me_3TFB-BD, and c) Me_3TFB-PA. Dashed lines in panel a indicate the characteristic stretching vibrations of the imine (1623 cm^{-1}) and carbonyl groups (1689 cm^{-1}), respectively.

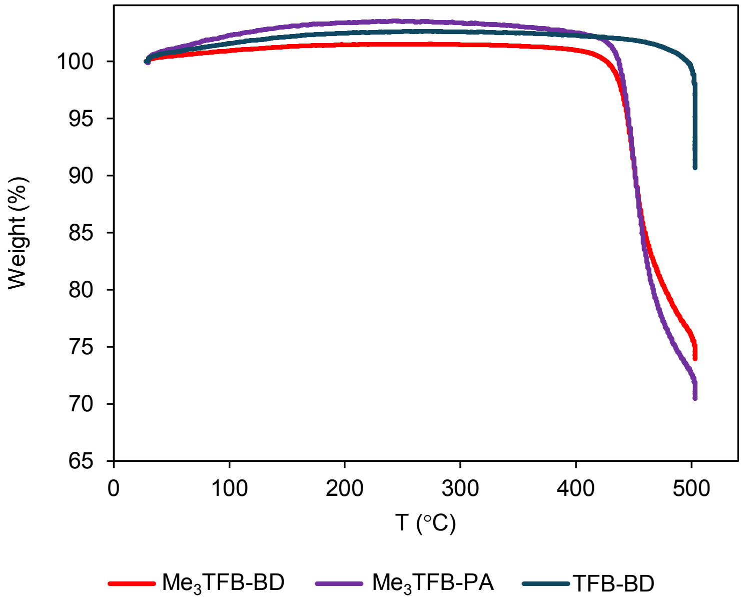

Figure S2. TGA plots of TFB-BD (green), Me_3TFB-BD (red), and Me_3TFB-PA (purple) used for IGC analysis. The COF powders were pre-dried at 120 °C to remove any remaining moisture. The steep vertical drop observed at T = 500 °C is due to an isothermal hold at this temperature of the TGA instrument, during which further thermal degradation of the COF occurred.

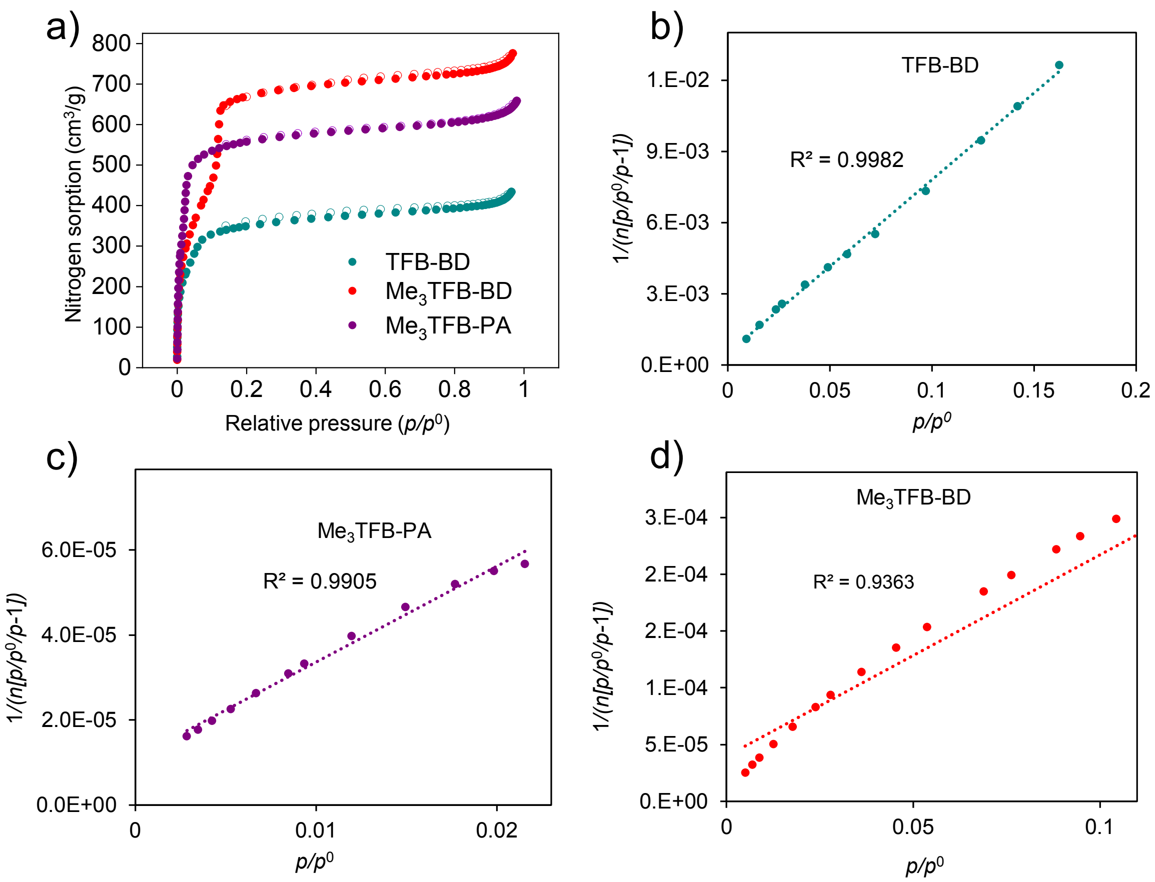

Figure S3. a) Sorption isotherms of TFB-BD, Me_3TFB-BD, and Me_3TFB-PA, where the closed symbols represent adsorption and desorption, respectively, and BET linearization plots for b)TFB-BD, c) Me_3TFB-BD, and d) Me_3TFB-PA.

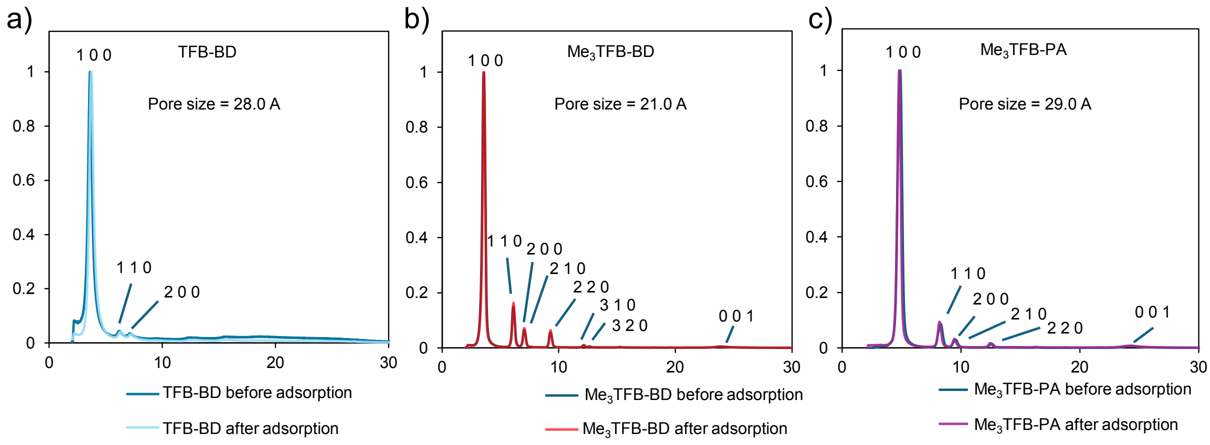

Figure S4. a) PXRD patterns of a) TFB-BD, b) Me_3TFB-BD, and c) Me_3TFB-PA. The peak positions were calculated using Miller indices.

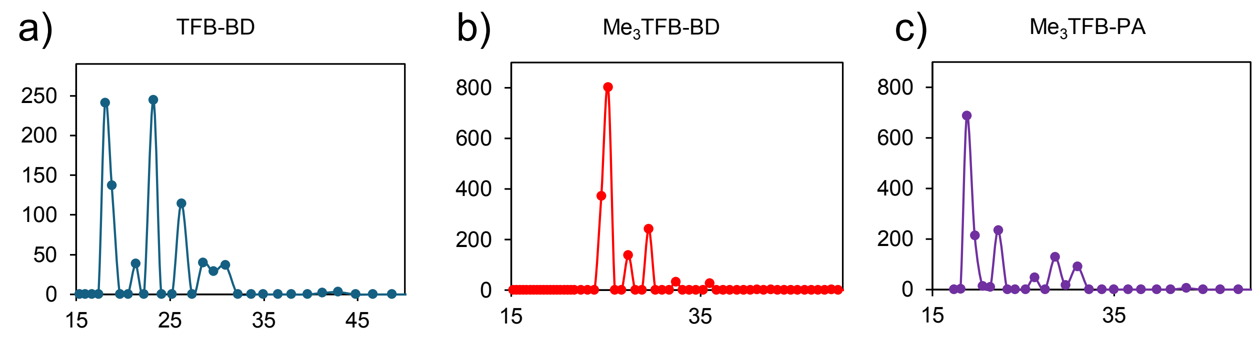

Figure S5. Pore-size distribution (PSD) plots of a) Me_3TFB-PA, b) TFB-BD, and c) Me_3TFB-BD. All PSD data was obtained from Figure S3 by calculating the pore size distributions using a cylindrical oxide model. Calculated average PSD values were found to be 1.9, 1.9 and 2.5 nm, respectively.

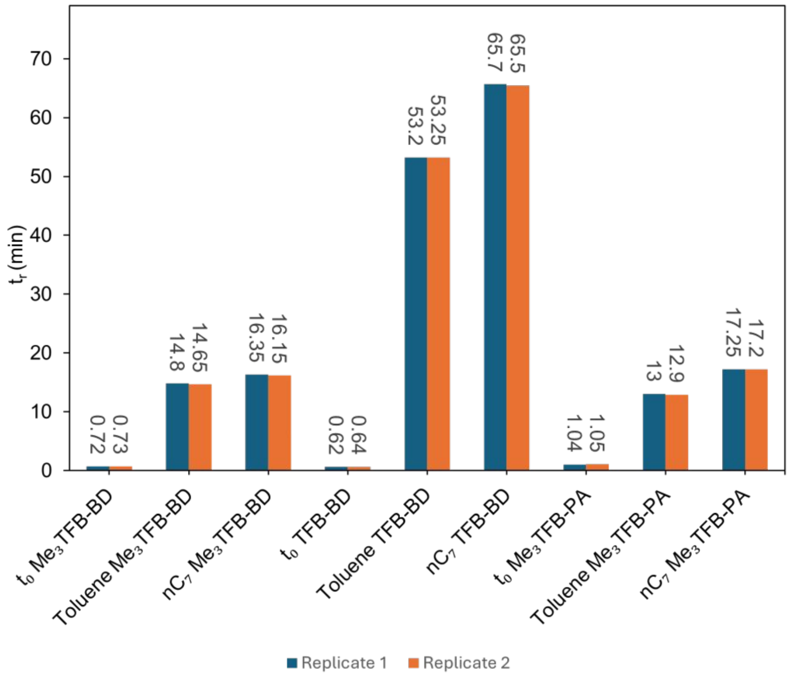

Figure S6: Retention time (t_R) data for duplicate injections of toluene, nC_7, and methane (t_0) onto TFB-BD, Me_3TFB-BD and Me_3TFB-PA at infinite dilution concentrations. Experiments were performed at T = 150 °C.

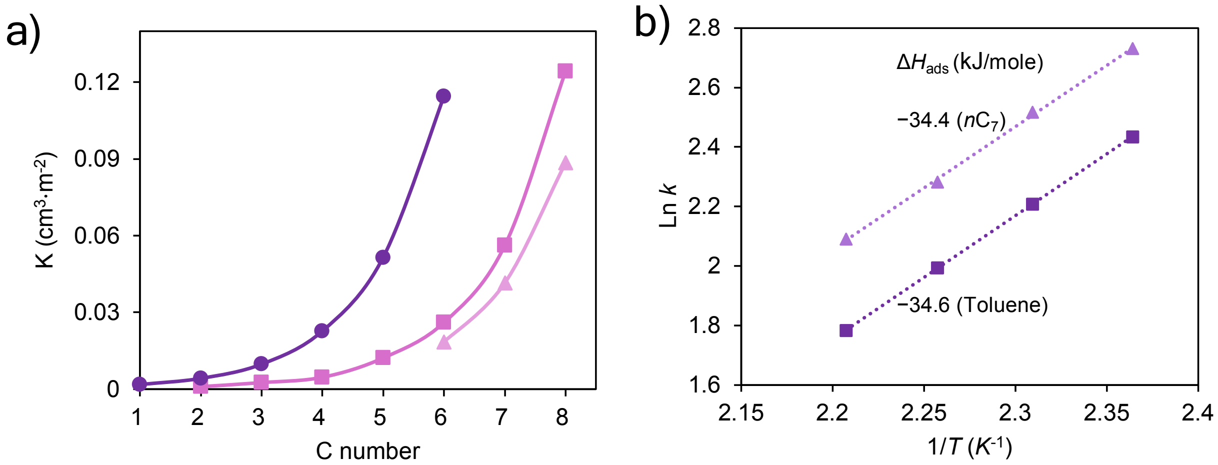

Figure S7. a) Partition coefficients of Me_3TFB-PA determined via IGC. Probe series are represented as follows: alkanes (●), alcohols (□), and alkylbenzenes (□). b) Enthalpy of adsorption for Me_3TFB-PA, based on injected probes *n*-heptane (*n*C_7, □) and toluene (□). Experimental *T* = 150-180 °C.

Table S1: Calculated Kovats Retention Indices for each COF, determined using **Equation 5**.

		**Retention indices**	
**Probe**	TFB-BD	Me_3TFB-BD	Me_3TFB-PA
C1-OH	0	-26.1	-35.6
C2-OH	0	-9.6	-18.4
C3-OH	0	-14.2	-24.7
C4-OH	0	-11.8	-26.9
C5-OH	0	-6.8	-26.1
Cyclohexane	0	2.5	-1.2
Methylcyclohexane	0	2.8	1.2
Benzene	0	0.2	-19.8
Toluene	0	8.3	-18.7
Ethylbenzene	0	9.8	-18.4
p-Xylene	0	-9.2	-22.4
Styrene	0	-32.9	-39.6
